# Cloning of *Vibrio cholerae* outer membrane protein W in *Pichia pastoris*

**Published:** 2013-09

**Authors:** Javad Alizadeh, Reza Ranjbar, Mehdi Kamali, Nima Farhadi, Amin Davari, Nourkhoda Sadeghifard

**Affiliations:** 1 Molecular Biology Research Center, Baqiyatallah University of Medical Sciences, Tehran, Iran; 2 Nanobiotechnology Research Center, Baqiyatallah University of Medical Sciences, Tehran, Iran; 3 Clinical Microbiology Research Center, Ilam University of Medical Sciences, Ilam, Iran

**Keywords:** *Vibrio cholerae*, cloning, outer membrane protein W, *Pichia pastoris*, PCR, Sequencing

## Abstract

**Background and Objective:**

The outer membrane protein W (*ompW*) of *Vibrio cholerae* is involved in stimulating the immune response via induction of protective immunity. It also plays an important role in bacterial pathogenesis by increasing the adaptability of pathogenic strains. In this study we aimed to clone *V. cholerae ompW* gene in the strain *X-33 of Pichia pastoris*.

**Materials and Methods:**

A gene encoding *ompW* was cloned into the Ppicza vector downstream of alcohol oxidase promoter. Then recombinant vector was transformed into the genome of the strain X-33 of *P. pastoris*. After growth of zeocin-resistant transformants, clones were selected and subsequently confirmed for cloning by PCR enzymatic digestion and sequencing.

**Results:**

PCR, enzymatic digestion and sequencing showed that the *ompW* gene was correctly cloned into *P. pastoris* genome.

**Conclusion:**

Results of our study showed that the methylotrophic yeast *P. pastoris* can be considered as an appropriate host instead of mammalian and prokaryotic systems for cloning of *ompW*. As far as data show, this is the first time that *ompW* of *V. cholera* is cloned into the methylotrophic *P. pastoris*.

## INTRODUCTION

*Vibrio cholerae* is the etiologic agent of the well-known diarrheal disease cholera, through intestinal colonization and elaboration of a potent enterotoxin. It is a Gram-negative bacterium which is classified into the γ-subdivision of *Proteobacteriaceae* family. Since the beginning of recent epidemic in 1991, over 700,000 cases of cholera have been reported just in the western hemisphere, and also over 100 cases were reported in the United States in 1992 (1). Cholera is an endemic infectious disease in many parts of Iran. The recent cholera outbreaks in Iran have mostly been caused by *V. cholerae* serotype Inaba (2–4).

O1 and O139 serogroups of *V. cholerae* which produce cholera toxin are associated with epidemic cholera, and all other members of the species are either nonpathogenic or just occasional pathogens (5). As for *V. parahaemolyticus*, there are some strains which are among the major causative agents of gastroenteritis (6).

Three crucial layers involved in the surface envelope of *V. cholera* include the cytoplasmic or inner membrane (IM), the outer membrane (OM) and the space between the IM and OM which known as periplasmic space (7). The phospholipids, LPS and proteins are the major components of OM by which OM is serving as a physical barrier between the bacterial body and its surroundings (8). OM also helps the organism to survive against materials such as bile salts. Omps are anchored to the membrane by the means of N-terminally linked lipids (7). One of these proteins, OmpW, is a 22kDa protein and belongs to a family of small Omps which are widespread in Gram-negative bacteria. It forms an 8-stranded beta-barrel with a long and narrow hydrophobic channel which is involved in osmoregulation and transportation of small hydrophobic molecules across the bacterial outer membrane (9). This protein is encoded by a gene that is located in the small chromosome (Chr II) of *V. cholera* (10). It is very immunogenic for immune system, so it can be considered as a dependent candidate to develop vaccines for prophylaxis purposes (9). *ompW* has been previously cloned and expressed in *E. coli* systems (7). In general, however, due to limitations and disadvantages of bacterial systems (11), yeasts have gained a lot of interest in cloning and expression of heterologous proteins (12).

Among eukaryotic systems in which scientists are recently more interested, the methylotrophic yeast, *P. pastoris* has been considered as an appropriate host for both small and large scale production of proteins (13). A number of properties make *P. pastoris* to be suited for expression of many simples and complex proteins. It has a high growth rates on simple and inexpensive media. This yeast has two alcohol oxidase genes named AOX1 and AOX2. These genes have a strongly inducible promoter and allow the yeast to use methanol as a carbon and energy source (14, 15).

In the present study, for the first time, we aimed to clone the *ompW* gene from *V. cholerae* serogroup *O1*, serotype *ogawa* in methylotrophic X-*33* strain of *P. pastoris*.

## MATERIALS AND METHODS

### Bacterial and yeast strains

*E. coli DH5a* and X-*33* strain of *P. pastoris* were provided from Pasteur Institute of Iran and National Institute of Genetic Engineering and Biotechnology, Tehran, respectively.

### Isolation of ompW gene

Bacterial genome was extracted from *V. cholerae* serogroup O1 serotype Ogawa using a DNA extraction kit (Fermentas, Germany).

### Primers and PCR condition

Forward and reverse primers to amplify the *ompW* gene were designed by DNASIS and Oligo software's. PCR reaction consisted of PCR buffer (2.5 µl), Mgcl_2_ (2.5 µl), dNTP (2 µl), Primers (Forward: ctccgaattcataatggctcaccaagaagg; Reverse: tcgcgggtaccttagaacttataaccacc) (1.5 µl of each) template DNA (2 µl), PFU DNA polymerase (0.6 µl) and DDW (up to 25 µl). The amplified products were separated on a 2% agarose gel in Tris-Borate-EDTA buffer (TBE×1) (pH 8.2) by electrophoresis at 90V for 1 h, then were stained with ethidium bromide and visualized under UV illumination. The expected amplified band of PCR was cut and purified using a PCR product purification Kit (Fermentas, Germany).

### Construction of recombinant vector

*ompW* gene with compatible ends engineered with *Eco*RI and *Kpn*I restriction enzymes was inserted into correct sites of the pPICZA vector which had also been cut with the same restriction enzymes. After ligation reaction, PCR amplification was performed and *ompW* gene was subsequently subjected to sequencing in recombinant vector to confirm its correct sequences.

### Transformation of recombinant plasmid into *E. coli* DH5α

Transformation of recombinant plasmids to electrocompetent *E. coli* DH5α cells was performed using electroporation protocol recommended by Bio-Rad Company (Laboratories, 1993, Gene Pulser Electro protocols). After electroporation, the bacterial suspension was incubated in a shaker incubator at 37°C for 1-1.5 hr to recover transformed cells using Super Optimal Broth (SOB). A microtube containing SOB solution was also incubated as control. The bacterial cells were centrifuged at 5000 rpm for 5 min. Then the supernatant was discarded and the pellet cells were cultured on LB agar containing zeocin as a resistance marker for the vector. The plates were then incubated at 37°C overnight.

### Confirmation of *E. coli* clones transformed with recombinant plasmid by PCR and enzymatic digestion

*E. coli* integrants were analyzed by PCR and enzymatic digestion to confirm the transformation of recombinant vector. Plasmid purification (Genejet plasmid Miniprep kit, Fermentas, Germany) was performed for *E. coli* clones grown on LB agar and purified plasmid was used as template in PCR reaction using of above mentioned *ompW* forward and reverse primers. PCR were performed in 25 µl solutions containing plasmid DNA (2 µl), primers (1.5 µl of each), deoxynucleotide triphosphates (2 µl), MgCl_2_ (25 mM), PCR buffer (2.5 µl) and *Taq* polymerase. The reaction initiated with a hot start at 94°C for 3 min, followed by 30 cycles of denaturation at 94°C for 1 min, annealing at 60°C for 1 min and extension at 72°C for 5 min. For the digestion reaction, recombinant plasmids were extracted (Gene Jet plasmid Miniprep kit, Fermentas, Germany) and double digestion reaction was accomplished in a reaction containing recombinant vector (8 µl), buffer (2 µl), *Eco*RI and *kpn*I (1 µl +1 µl) and DDW (up to 20 µl).

### Transformation of linearized plasmid into electrocompetent yeast cells

Recombinant pPICZA plasmid was digested with *sac*II restriction enzyme in a reaction containing recombinant vector (15 µl), buffer (2 µl), *sacII* (1.5 µl), DDW (up to 20 µl). Linearized form of recombinant plasmids was transformed into electro competent yeast cells. Electroporated yeast cells were then cultured on Yeast Extract Peptone Dextrose (YEPD) medium plates and incubated at 30°C for 72-94 hr. Clones grown on plates were passaged into falcons to prepare stocks and for genome extraction purposes.

### PCR analysis of *Pichia integrants*

The *Pichia* integrants were analyzed by PCR to confirm the integration of recombinant vector with *ompW* gene into *Pichia* genome. To amplify *ompW* gene for cloning into a the vector, genomic DNA was extracted (16) and used as template. PCR reaction consists of *Taq* polymerase (0.5 µl), yeast genomic DNA (2 µl), primers (1.5 µl of each), dNTP (2 µl), MgCl_2_ (1.5 µl) and PCR buffer (2.5 µl) in a total volume of 25 µl. The amplification cycle was initiated by a hot start at 94°C for 3 min followed by 30 cycles of denaturation at 94°C for 1 min, annealing at 60°C for 1 min and extension at 72°C for 5 min.

### Sequencing analysis of *pichia integrants*

The genomic DNA was extracted from transformed yeasts then used as template for PCR reaction according to previously described condition using AOX1 forward and reverse primers and pfu DNA polymerase. PCR products were purified as recommended by the company (PCR product purification Kit, Fermentas, Germany). The recombinant plasmids were also extracted according to previously described method. Finally both of the genomic PCR products and recombinant plasmids were subjected to sequencing.

## RESULT

### Isolation of *ompW* gene and transformation of linearized plasmid into electrocompetent yeast cells

**Fig. 1:**
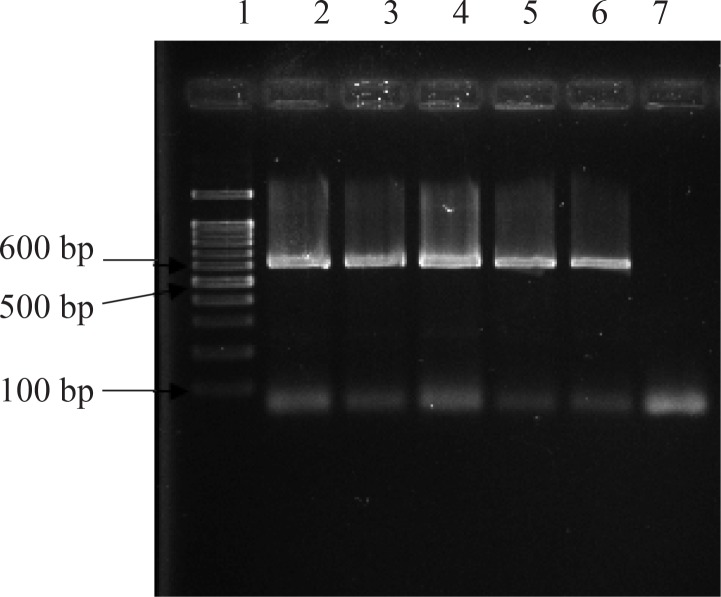
PCR of bacterial genome (*Vibrio cholera*) to amplify *ompW* gene. Lane 1, molecular weight markers, Lanes 2, 3, 4, 5, 6 (respectively clones 1-5) Lane 7, negative control with no template

The expected band of *ompW* gene (600 bp) was amplified by PCR reaction (Fig. 1). Transformants were appeared on LB-agar media (zeocin included) 3 to 4 days after transformation. Five clones (1-5) were selected for analysis by PCR for further confirmation of the integration of gene into AOX1 gene.

### PCR analysis of *Pichia integrants*

**Fig. 2:**
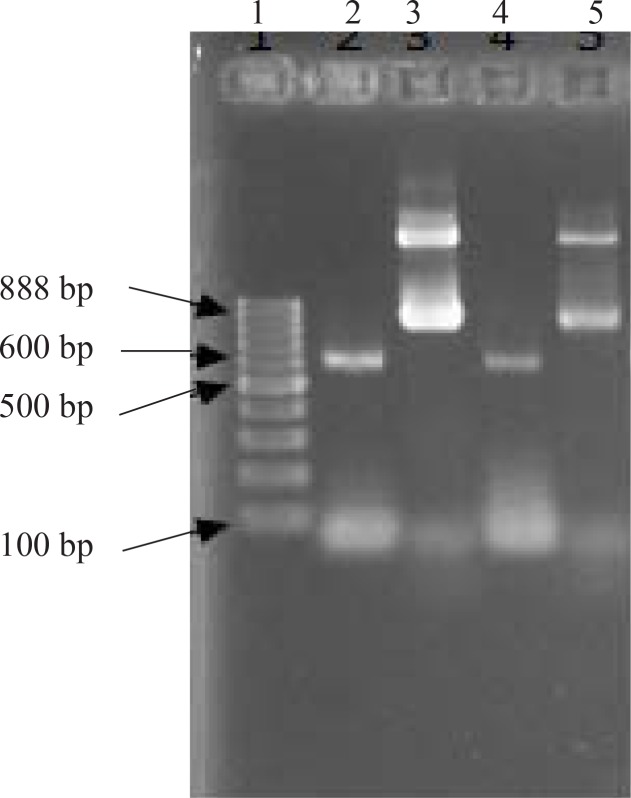
PCR analysis of clones 1 and 5 Lane 1, molecular weight markers Lane 2, clone1 with ompW primers, Lane 3, clone1 with AOX1 primers, Lane 4, clone5 with ompW primers, Lane 5, clone5 with AOX1 primers. The band upper to 888bp in lanes 3 and 5 is a site in yeast chromosome which has homology to our primers.

Five integrant clones were analyzed by PCR in optimized situation (Fig. 2). From all 5 clones, a band of 888 bp (consist of a 600 bp part for *ompW* and another 288 bp part for pPICZA vector), was produced indicating that target gene was correctly integrated into AOX1 gene of yeast genome. In two clones, the above mentioned band was sharper than others, therefore these clones (1 and 5) were chosen for the next steps of study (Fig. 2).

### Sequencing analysis of *Pichia integrants* and recombinant plasmids

**Fig. 3:**
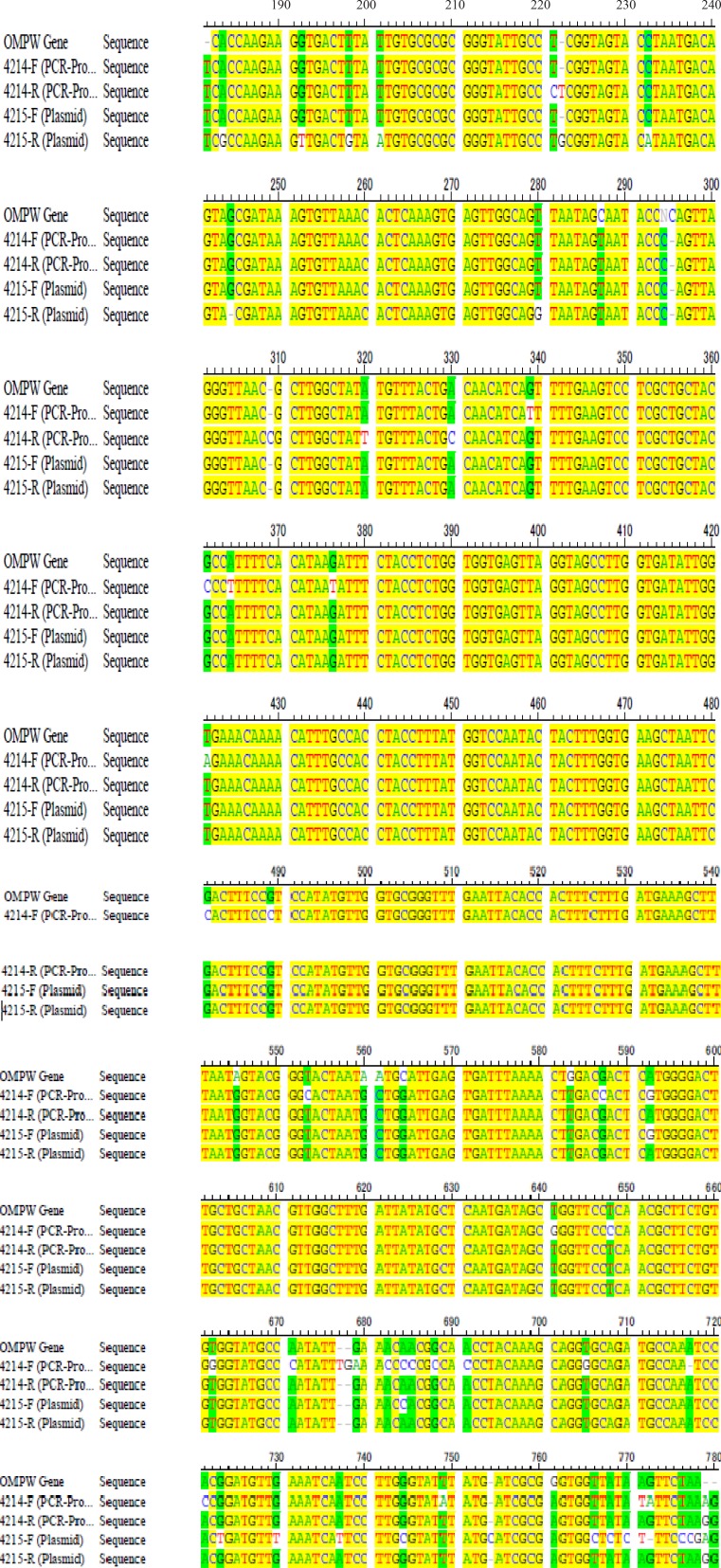
Analysis of sequencing data from both cloned plasmids and PCR-product by DNA SIS software.

Results obtained from sequencing indicated that *ompW* gene was correctly cloned into yeast genome (Fig. 3).

## DISCUSSION

In this study, *ompW* was successfully cloned in a yeast host *P. pastoris*. This protein is involved in pathogenicity of *V. cholerae* through osmoregulation and transportation of small hydrophobic molecules across the bacterial outer membrane. It also functions as a facilitating protein to CTX toxin. Considering these important functions, it has been greatly considered for its protective potential as an immunogenic candidate (7). In the most previous studies, most proteins have been cloned and expressed in *E. coli* and *Saccharomyces cerevisiae* systems (11, 17, 18).

Because of specific cytoplasm environment of the prokaryotic organisms, generally, these systems have been unsuccessful in cloning and expression of heterologous proteins. Therefore, there is a great need to further process the recombinant proteins and also to secrete these recombinant proteins out from cytoplasm (17). In spite of efforts and studies that have been done to overcome these bottlenecks of prokaryotic systems, there are still problems that researchers should deal with them (19, 20). The Chinese Hamster Ovary (CHO) cells have also been evaluated as an appropriate host system for recombinant protein production, but there are some disadvantages such as probability of viral and prion contamination, expensive cell culture media, and purification problems which are remarkable problems of mammalian host systems (21).

*Saccharomyces cerevisiae* has been used as a host for expression of different kinds of heterogeneous proteins (15). In spite of its advantages as a host, in some studies, secretion of recombinant proteins into the culture medium of *S. cerevisiae* has not been observed (22). This state has to be followed by purification of recombinant proteins from cell debris which is not very cost-effective.

Reiser and his colleagues showed that proteins involved in amino acid or nucleotide acid metabolism expressed in *Saccharomyces cerevisiae* are hyper-glycosylated (23). Romanos and his colleagues observed that some amount of recombinant protein which was *Bordetella pertusis* practin was retained in periplasmic space of yeast *S. cerevisiae* and due to this problem, protein is degraded to some degree (24).

Taking into consideration the disadvantages of *S. cerevisiae*, we used *P. pastoris* as a host in our study. *P. pastoris* has some advantages over other hosts. It has a high growth rate and is able to grow on a simple, inexpensive medium. It is suitable for both small and large scale production because it can grow in either shake flasks or a fermenter (25). The major plus point of *Pichia* over *E. coli* is that *Pichia* is able to produce disulfide bonds and glycosylations in proteins. *Pichia is* a system that is cheap to set up and maintain and can be easily grown in cell suspension in solutions with reasonably strong methanol that would kill most micro-organisms. Moreover, it can grow to very high cell densities under ideal conditions (26).

Different studies have shown that *P. pastoris* is a good host and has the capability of cloning and expressing various numbers of heterologous proteins. Hohenblum and his colleagues postulated that tripsinogen cloned and expressed in *P. pastoris* has the correct number of disulphide bonds (12). In a study conducted by Majidzadeh and his colleagues, *P. pastoris* was used as a host for cloning and expressing of full-length human tissue plasminogen activator (27). In another study, Clare and his colleagues cloned and expressed the tetanus toxin in *P. pastoris* and concluded that the amount of exotoxin or LPS was very low in final product in comparison to bacterial system (28). In this study, we took advantage of yeast *P. pastoris* as cloning host which bypasses all the problems of other hosts aforementioned. PCR and double digest reactions indicated that the *ompW* gene was cloned properly into the yeast and sequencing confirmed our results. It should be noted that we could not investigate the expression status of the cloned gene in this stage, however we hope that the results obtained from our work will possibly provide a baseline for future related studies.

In conclusion, the *ompW* of *V. cholerae* was properly cloned into methylotrophic yeast *P. pastoris*. Results of our study showed that the methylotrophic yeast *Pichia pastoris* can be considered as an appropriate host instead of mammalian and prokaryotic systems for cloning of *ompW*.
